# High-density genetic linkage map construction and cane cold hardiness QTL mapping for Vitis based on restriction site-associated DNA sequencing

**DOI:** 10.1186/s12864-020-06836-z

**Published:** 2020-06-22

**Authors:** Kai Su, Huiyang Xing, Yinshan Guo, Fangyuan Zhao, Zhendong Liu, Kun Li, Yuanyuan Li, Xiuwu Guo

**Affiliations:** 1 College of Horticulture, Shenyang Agricultural University, Shenyang, P.R. China; 2 National & Local Joint Engineering Research Center of Northern Horticultural Facilities Design & Application Technology, Shenyang, P.R. China; 3 College of Horticulture Science and Engineering, Shandong Agricultural University, Shandong, P.R. China

**Keywords:** Grapevine, Single-nucleotide polymorphism marker, Restriction-site associated DNA sequencing, Cold hardiness, Quantitative trait loci mapping, Molecular breeding

## Abstract

**Background:**

Cold hardiness is an important agronomic trait and can significantly affect grape production and quality. Until now, there are no reports focusing on cold hardiness quantitative trait loci (QTL) mapping. In this study, grapevine interspecific hybridisation was carried out with the maternal parent ‘Cabernet sauvignon’ and paternal parent ‘Zuoyouhong’. A total of 181 hybrid offspring and their parents were used as samples for restriction-site associated DNA sequencing (RAD). Grapevine cane phloem and xylem cold hardiness of the experimental material was detected using the low-temperature exotherm method in 2016, 2017 and 2018. QTL mapping was then conducted based on the integrated map.

**Results:**

We constructed a high-density genetic linkage map with 16,076, 11,643, and 25,917 single-nucleotide polymorphism (SNP) markers anchored in the maternal, paternal, and integrated maps, respectively. The average genetic distances of adjacent markers in the maps were 0.65 cM, 0.77 cM, and 0.41 cM, respectively. Colinearity analysis was conducted by comparison with the grape reference genome and showed good performance. Six QTLs were identified based on the phenotypic data of 3 years and they were mapped on linkage group (LG) 2, LG3, and LG15. Based on QTL results, candidate genes which may be involved in grapevine cold hardiness were selected.

**Conclusions:**

High-density linkage maps can facilitate grapevine fine QTL mapping, genome comparison, and sequence assembly. The cold hardiness QTL mapping and candidate gene discovery performed in this study provide an important reference for molecular-assisted selection in grapevine cold hardiness breeding.

## Background

Grapevine (2n = 38) is perennial deciduous vine fruit liana which belongs to the genus Vitis of the Vitaceae family and has high economic and social values. In 2016, the cultivated area of grapevine in China was 847,000 ha with a total production of 13.1 million tons, accounting for 15.1% of the world’s grape output (http://www.fao.org/faostat/zh/#home). *Vitis vinifera* L*.* is the major cultivated grapevine species in China as table grapes and is a preferred raw material for making vine. *Vitis vinifera* L. is originated in the Mediterranean region where the climate is hot and dry in the summer and warm and rainy in the winter. However, China is located in typical continental monsoon climate region where cold and dry in winter. The annual lowest temperature of most grape-producing regions in China below − 15 °C, thus it is necessary for grapevine to be buried with soil to resist the cold environment. This strategy greatly increases management costs and can also lead to the damage to the grapevine and soil structure, causing dust storms and soil erosion.

Plants usually undergo cold stress when temperatures fall below − 10 °C. Injury is associated with a complex array of cellular dysfunctions, and symptoms include loss of vigour, wilting, chlorosis, sterility, and even death [1]. Wild Vitis species such as North American (*V. riparia* Michx., *V. labrusca* L., *V. rupestris* Scheele.) and Asian (*V. amurensis* Rupr.) species show significant cold hardiness, tolerating − 30 °C or even lower [2]. These wild Vitis species have been used in grapevine breeding programs for the selection of new cold hardiness cultivars. However, grapevine is a highly heterozygous species with a long developmental period and complex genetic background [3]. An alternative strategy is cultivating cold hardiness resistance cultivars through traditional crossbreeding. While traditional crossbreeding was lengthy and had lower breeding efficiency in the past. In recent years, marker-assisted selection (MAS) was widely used for the research of grape breeding based on genetic linkage map construction and QTL mapping. This strategy will make grapevine breeding more efficient and precise [3–6].

The strategy of genetic map construction in fruit trees was based on the theory of double pseudotest cross, and most of the materials used were F1 hybrid populations [7]. Single-nucleotide polymorphisms (SNPs) are codominant marker types with high genetic stability and available for their accurate detection. In recent years, with the development of next-generation sequencing (NGS) technology, simplified genome sequencing based on this technology has been widely used for identifying SNP markers and constructing grapevine genetic maps [5, 6, 8–13]. As one of the major simplified genome sequencing technologies, restriction-site associated DNA sequencing (RAD) has been widely used in genetic map construction for grapevine and other species [9, 12, 14–21]. Until now, many QTLs and SNP markers related to important quantitative traits of grapevine were identified by using biparental mapping and genome-wide association study (GWAS). They were used to investigate diseases resistance genes related to powdery mildew [10, 22–25], downy mildew [6, 25–28], Pierce’s disease [29–31], grape phylloxera [5] and phomopsis disease [32]. They have also been used to identified genes related to a series of agronomic traits such as berry size and weight, firmness, sugars and acids content, color, muscat flavor [33–44], architecture of the grapevine cluster [45], fruit yield and quality [46, 47], seed weight and number [48], flower sex [26, 30, 49, 50], fertility [51], inflorescence morphology [26], timing and duration of flowering and of veraison [34, 52].

No studies have focused on QTL mapping of grape cane cold hardiness. In this study, after years of field observation, *V. vinifera* L. cultivar ‘Cabernet sauvignon’ showed weak cold hardiness and cultivar ‘Zuoyouhong’ which was obtained by crossing of *V. vinifera* × *V. amurensis* showed high cold hardiness. Interspecific hybridization was then conducted and ‘Cabernet sauvignon’ was used as the maternal parent and ‘Zuoyouhong’ was used as the paternal parent. RAD sequencing and marker development were conducted based on two parents and 181 hybrid offspring. A high-density linkage map was constructed, and cane cold hardiness QTL mapping was carried out considering with 3 years of cold hardiness phenotype data. This study will provide a foundation for MAS in grapevine cane cold hardiness breeding.

## Results

### Cane cold hardiness analysis

**Fig. 1:**
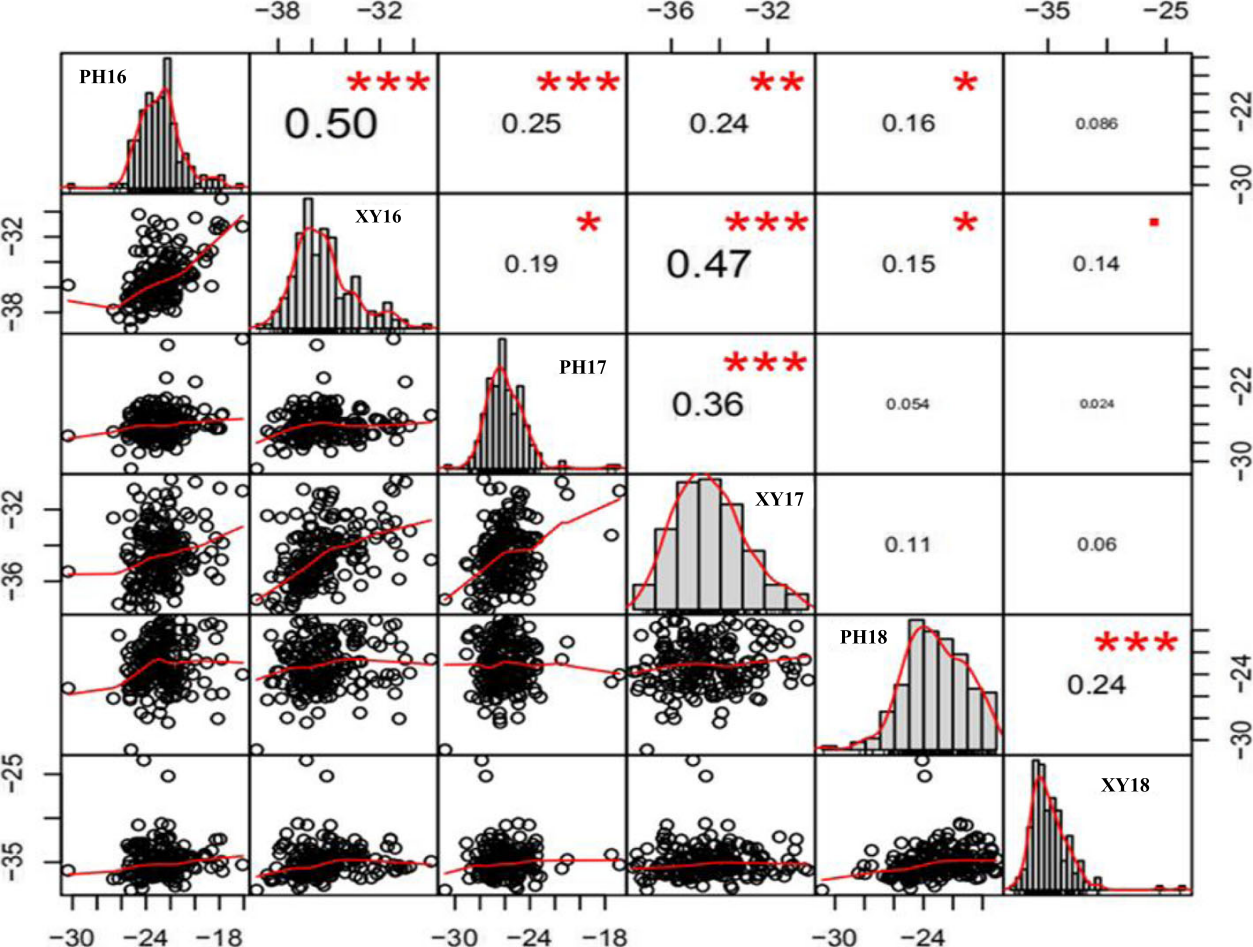
Correlations analysis of phenotypic data between different years. “*”, “**” and “***” represent the significant level at *P* < 0.01, 0.005 and 0.001

Grapevine cane samples from 2016, 2017, and 2018 of the two parents and 181 individuals were identified by differential thermal analysis. Lethal temperature of phloem (LTP) and lethal temperature of xylem (LTX) during these 3 years were named as PH16, XY16, PH17, XY17, PH18, and XY18 (Additional file 1: Data S1). These values (mean value of three replicates per genotype) showed continuous variation, indicating the grapevine cold hardiness as a typical quantitative trait controlled by polygenes. Based on Shapiro-Wilk tests, these values from LTP and LTX during 3 years showed a non-normal distribution (*P* < 0.05). The correlation coefficients of LTP and LTX values in the same year were significant at *P* < 0.001 and ranged from 0.24 to 0.50. For LTP and LTX in three different years, PH16, PH17, and XY16, XY17 showed significance at *P* < 0.001 and ranged from 0.25 to 0.47, PH16 and PH18 showed significance at *P* < 0.05 with a correlation coefficient of 0.16. In addition, PH16 and XY17, XY16 and PH17, and XY16 and PH18 also showed a significant correlation at *P* < 0.05 and *P* < 0.005 (Fig. 1). The equation for the broad sense heritability (*H*^*2*^) calculation was *H*^*2*^ = V_G_/(V_G_ + V_E_), V_G_ and V_E_ represent genetic variance and environmental variance, respectively. The traits datasets we collected has 181 lines and they were evaluated in 3 environments and 3 replications in 3 years, the genetic variance and *H*^*2*^ were estimated by using “mmer” function in *sommer* R packages executed liner mixed models [53, 54]. The year-to-year variance for LTP was and LTX were 3.08 and 2.57. *H*^*2*^ of LTP was 0.42, and the *H*^*2*^ of LTX was 0.56.

### Raw data analysis and SNP marker development

In total, 322.68 Gb of data were obtained from the two parents and 181 hybrid offspring based on RAD sequencing; 1,010,172,055 clean reads were obtained by filtering the original data, among which 46,762,423 were from the maternal parent ‘Cabernet sauvignon’ and 36,408,094 were from paternal parent ‘Zuoyouhong’. Clean read number distributions of the 181 hybrid offspring shown in Additional file 2: Fig. S1. In the filtered data, the GC content and Q30 of the maternal parent were 36.44 and 91.64%, paternal parent were 37.97 and 93.80%. Sequencing depth can affect accuracy of mutation detection. In this study, the average sequencing depths of ‘Cabernet sauvignon’ and ‘Zuoyouhong’ were 24.01 and 19.40, respectively, the sequencing depth distribution of the hybrid offspring is shown in Additional file 2: Fig. S1.

**Fig. 2:**
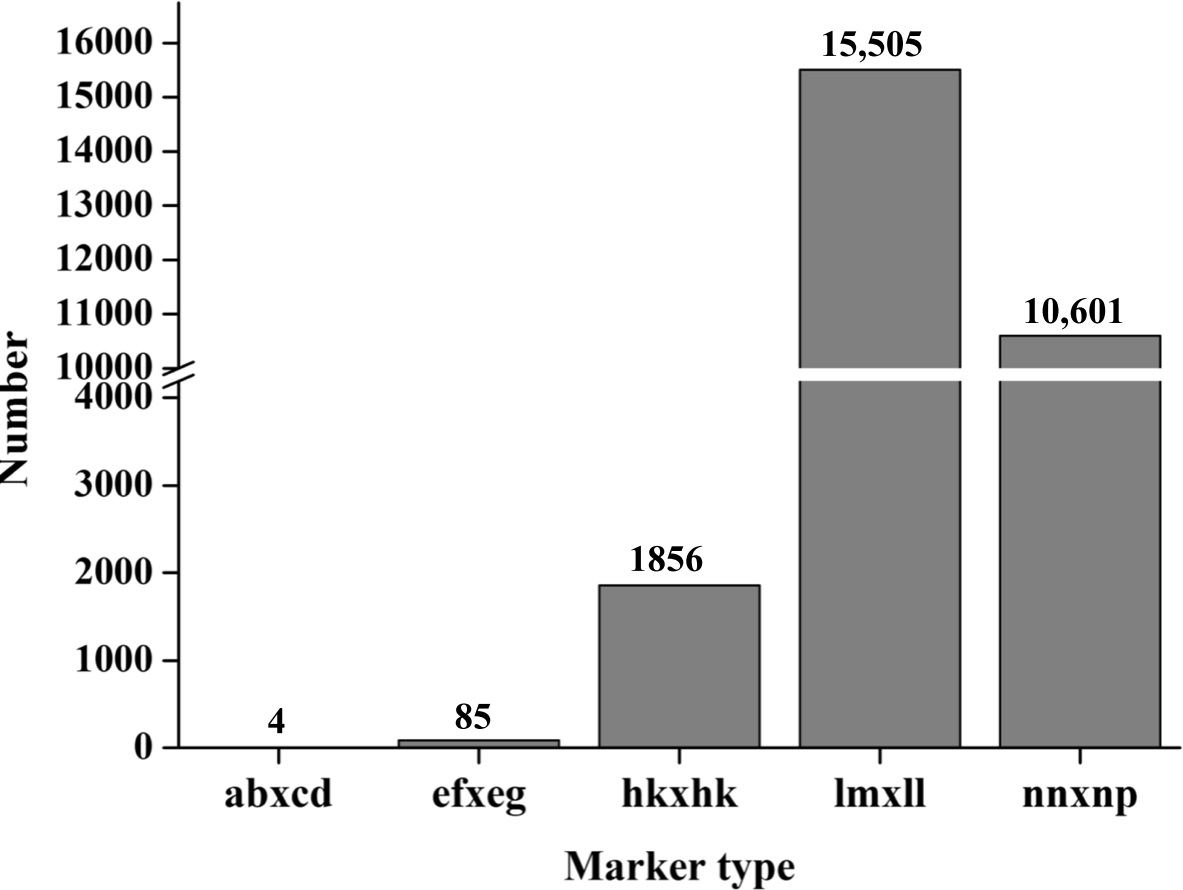
Number of different genotype markers. lm × ll represent the markers used for female map construction and the order was male×female, nn × np represent the markers used for male map construction and the order was female×male, ab×cd, ef × eg and hk × hk represent the markers contained by both of the parents

In total of 56,779 markers were called in this study, among them, 6971 were monomorphic marker. A Chi-square test (*p* < 0.01) was conducted for these polymorphism markers and 14,927 distorted markers were then removed. After standard filtering, 28,051 markers were obtained and used to construct genetic linkage maps (Table 1). Of the 28,051 SNP markers, 26,106 were homozygous for one parent and heterozygous for the other (15,505 for lm × ll and 10,601for nn × np), constituting 93.1% of all selected SNP markers. The remaining 1945 markers were constituted by three different types, including ab × cd (4), ef × eg (85), and hk × hk (1856) (Fig. 2), these markers were contained in both the female and male maps.

**Table 1:** Number statistics analysis of different marker category

Category	Number
Original number of called markers	56,779
Monomorphic marker	6971
Distorted marker	14,927
Markers on the genetic map	28,051

### Genetic linkage map construction

The retained 28,051 markers were assigned to 19 linkage groups, finally, 25,917 were anchored on the genetic map at Logarithm of odds (LOD) score thresholds ≥7 (Table 2, Additional file 3: Data S2) and the Mendelian segregation and depth of each marker is shown in Additional file 4: Data S3. The Kosambi function was used to estimate genetic map distances. For ‘Cabernet sauvignon’, 16,076 SNP makers were distributed in 19 linkage groups with a total map length of 1548.11 cM. Among the 19 linkage maps, the shortest was LG11 with a genetic length of 53.72 cM and the longest was LG14 with a genetic length of 120.65 cM. Marker number in each linkage group ranged from 439 to 1715, LG2 contained the smallest number of markers and LG14 contained the largest number of markers (Table 2, Additional file 5: Data S4). In our study, many markers in the female map were anchored in the same genetic position, and we conducted analysis to determine these markers by generating bin markers. Each bin marker represents a unique position. For the female map, 2384 bin markers were obtained (Additional file 6: Data S5). The average genetic distance of adjacent bin markers in the 19 linkage groups was 0.65 cM. The longest average genetic distance of an adjacent marker was observed in LG10 with a length of 1.20 cM, whereas the shortest were found in LG13 with a length of 0.5 cM. The largest gap for this map was contained in LG1 with the distance 9.73 cM. Besides LG1, LG2, LG3, LG5, LG8, and LG10, the percentage of Gap ≤5 cM (gap less than or equal to 5 cM) checked in the other linkage groups reached 100% (Table 3, Additional file 7: Fig. S2).

**Table 2:** Marker distribution and total genetic length of 19 linkage groups

Linkage group ID	Maker Number			Genetic distance (cM)		
	Female Map	Male map	Integrated map	Female Map	Male map	Integrated map
LG1	775	477	1209	98.43	99.95	99.20
LG2	439	570	985	70.88	84.84	78.97
LG3	466	444	896	81.29	69.94	77.63
LG4	868	642	1415	78.29	137.36	109.78
LG5	776	871	1585	82.45	89.30	86.71
LG6	668	587	1117	58.01	79.03	69.36
LG7	1151	524	1584	91.45	103.33	106.52
LG8	571	411	955	86.19	102.77	154.17
LG9	644	567	1173	80.04	79.25	79.91
LG10	743	452	1065	62.59	52.58	75.82
LG11	764	679	1288	53.72	72.39	64.8
LG12	1281	604	1728	97.75	91.40	96.82
LG13	1357	515	1707	89.59	100.60	95.37
LG14	1715	866	2381	120.65	157.51	147.20
LG15	889	637	1362	75.26	102.39	88.83
LG16	633	643	1251	72.58	81.49	77.54
LG17	668	690	1173	58.39	63.37	61.07
LG18	822	799	1602	108.49	135.81	124.11
LG19	846	665	1441	82.06	87.9	86.67
Total	16,076	11,643	25,917	1548.11	1791.21	1780.48

**Table 3:** Genetic distance of adjacent markers in 19 linkage groups

Linkage group ID	Average genetic distance (cM)			Percentage of Gap≤5 cM(Max Gap)		
	Female Map	Male map	Integrated map	Female Map	Male map	Integrated map
LG1	0.72	0.85	0.41	99.87%(9.73)	99.58%(8.40)	100.00%(2.97)
LG2	0.84	0.81	0.44	99.77%(5.24)	99.82%(7.22)	99.90%(5.06)
LG3	0.82	0.93	0.46	99.57%(8.32)	99.55%(7.11)	100.00%(2.93)
LG4	0.67	0.95	0.46	100.00%(3.43)	99.69%(5.67)	100.00%(3.07)
LG5	0.70	0.63	0.36	99.87%(5.85)	100.00%(3.43)	100.00%(1.94)
LG6	0.60	0.79	0.37	100.00%(2.26)	100.00%(4.03)	100.00%(2.14)
LG7	0.62	1.04	0.48	100.00%(2.72)	99.43%(15.22)	99.94%(9.73)
LG8	0.75	0.88	0.70	99.65%(8.40)	99.51%(11.01)	99.69%(9.49)
LG9	0.68	0.71	0.37	100.00%(2.76)	100.00%(3.43)	100.00%(1.77)
LG10	1.20	0.60	0.57	99.60%(10.30)	100.00%(2.84)	99.72%(11.3)
LG11	0.65	0.78	0.39	100.00%(4.03)	99.85%(17.72)	100.00%(4.21)
LG12	0.52	0.66	0.31	100.00%(1.69)	100.00%(3.43)	100.00%(1.69)
LG13	0.50	0.94	0.35	100.00%(2.26)	99.81%(14.66)	100.00%(3.56)
LG14	0.56	0.77	0.39	100.00%(2.26)	99.88%(10.03)	99.96%(9.22)
LG15	0.55	0.65	0.33	100.00%(2.26)	100.00%(3.43)	100.00%(1.68)
LG16	0.59	0.62	0.32	100.00%(4.03)	100.00%(2.84)	100.00%(2.20)
LG17	0.61	0.60	0.36	100.00%(4.03)	100.00%(2.84)	100.00%(2.01)
LG18	0.74	0.78	0.40	100.00%(3.43)	99.87%(7.25)	100.00%(2.75)
LG19	0.68	0.87	0.41	100.00%(2.84)	99.85%(28.19)	100.00%(3.15)
Average	0.68	0.78	0.41			

A total of 11,643 SNP markers were anchored into 19 linkage groups of the paternal parent with a total genetic length of 1791.21 cM. The genetic length of each linkage group ranged from 52.58 to 157.51 cM. The longest was LG14 and shortest was LG10. Marker number in each linkage group ranged from 411 to 871; LG8 contains the smallest number and LG5 contained the largest number (Table 2, Additional file 5: Data S4). Finally, a total of 2330 bin markers were generated (Additional file 6: Data S5), and the average genetic distance between adjacent markers in the 19 linkage groups was 0.77 cM. The longest one was LG7 with genetic lengths of 1.04 cM, and the shortest ones were LG10 and LG17 with genetic lengths of 0.60 cM. For the male map, nearly half of the linkage groups contained the regions of Gap > 5 cM, and the largest gap for this map was contained in LG19 with the distance 28.19 cM (Table 3, Additional file 8: Fig. S3).

**Fig. 3:**
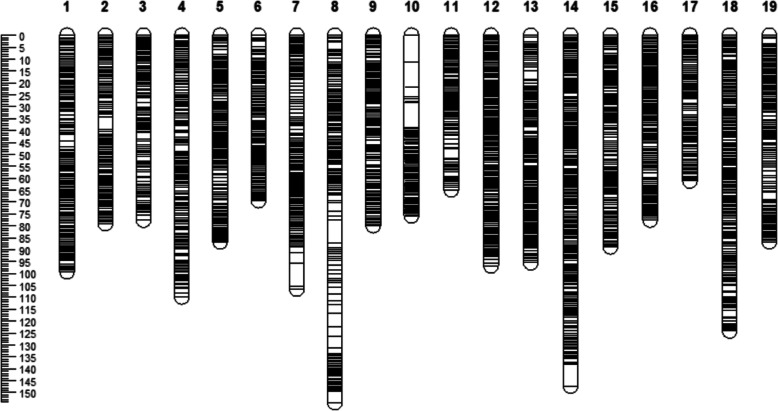
Marker distribution and genetic length of integrated map. Centimorgans (cM) indicated the genetic length of vertical scale. Black lines represent mapped markers. LG1–19 represents corresponding linkage groups

The integrated map contained 25,917 SNP markers with a total genetic length of 1780.48 cM. The shortest linkage group was LG17 and longest was LG8 with genetic lengths of 61.07 and 154.17 cM. Among the 19 linkage groups, LG14 contained the largest SNP number of 2381 and LG3 contained the smallest number of 896 (Table 2, Additional file 5: Data S4). A total of 4383 bin markers were generated (Additional file 6: Data S5), and the average genetic distance between adjacent bin markers in the 19 linkage groups was 0.41 cM. The shortest genetic distance was found in LG12 with a value of 0.31 cM, whereas the longest was found in LG8 with a value of 0.70 cM. Additionally, 8 Gap > 5 cM regions were found in LG2, LG7, LG8, LG10, and LG14, the largest gap was contained in LG10 with 11.3 cM (Table 3 and Fig. 3).

### QTL mapping and candidate genes involved in grapevine cold hardiness

**Fig. 4:**
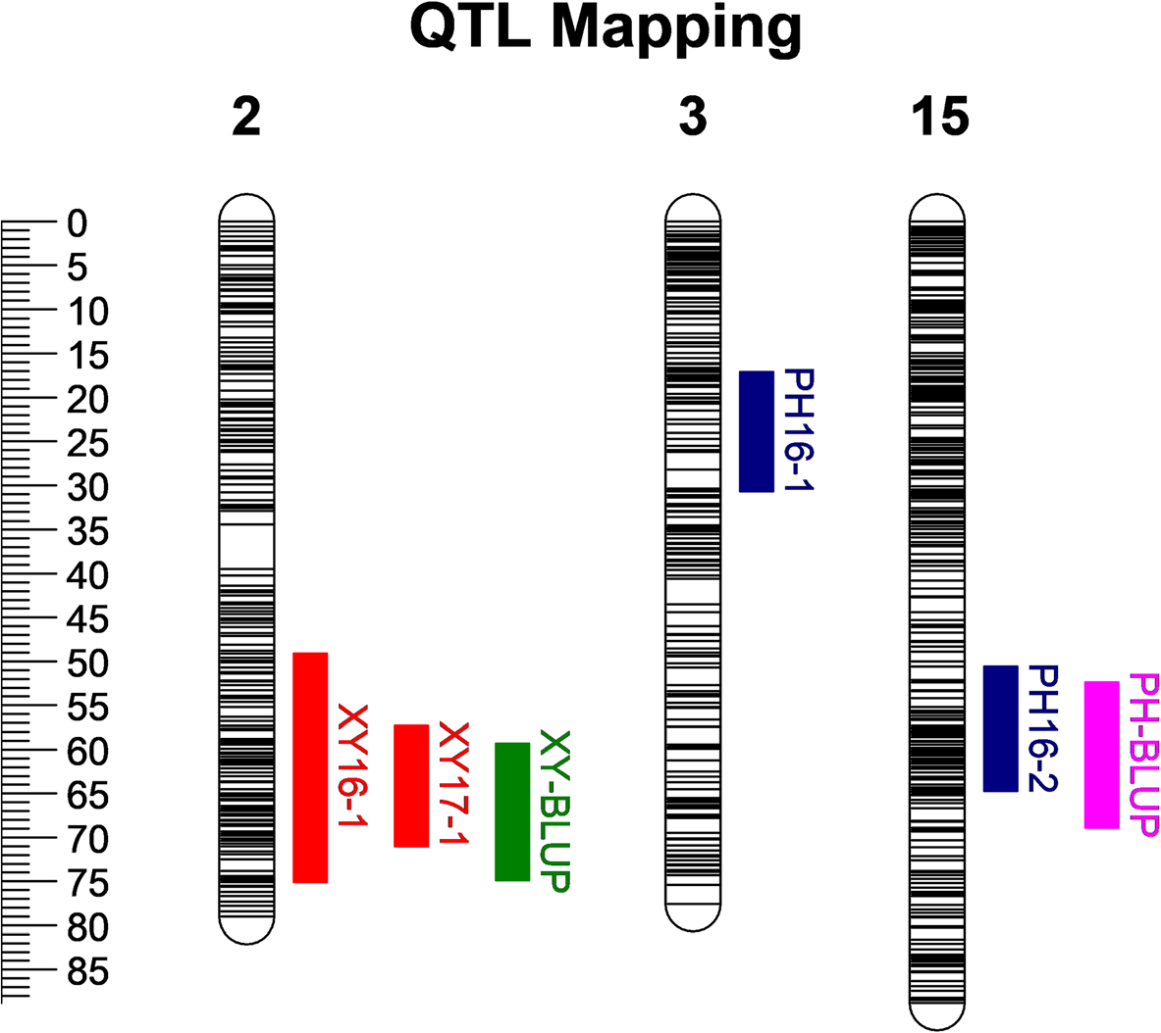
QTL mapping of grapevine cane cold hardiness. Blue color represents the confidence interval of grapevine phloem; red color represents the confidence interval of grapevine xylem; pink color represent the confidence interval of QTL mapping based on phloem BLUP values; green color represent the confidence interval of QTL mapping based on xylem BLUP values

In this study, we conducted QTL mapping for the LTP and LTX during 3 years based on the integrated map. The outliers of the phenotypic value including line1 in PH16, line 85 in XY16, line 139, 149, 156 and 168 in PH17 and line 59 and 121 in XY18 were removed prior to QTL mapping. For the LTP, two major QTLs were identified on LG3 and LG15, corresponding to the trait of PH16. The confidence intervals of these two QTLs were 17.11 cM–30.73 cM and 50.56 cM–64.66 cM, Each QTL explained 8.47–8.52% of the phenotypic variation (*R*^*2*^) (Table 4 and Fig. 4). For the LTX, two QTLs were identified on LG 2 in the year of 2016 and 2017. The confidence intervals of these two QTLs were 49.13 cM–75.07 cM and 57.29 cM–70.99 cM. The phenotypic variation they explained was 8.34 and 11.73%, respectively (Table 5 and Fig. 4).

**Table 4:** QTL mapping for lethal temperature of phloem based on integrated map

Traits	LG	Peak LOD	Co-segregated marker	Peak Location (cM)	*R*^*2*^(%)	Confidence interval (cM)
PH16	3	3.21	chr3_7,621,469	17.11	8.47	17.11–30.73
PH16	15	3.23	chr15_15,252,067	52.42	8.52	50.56–64.66
BLUP	15	3.87	chr15_16601112	61.95	7.33	52.42–68.94

*R*^*2*^ represents the individual contribution of one QTL to the variation in cold hardiness

**Table 5:** QTL mapping for lethal temperature of xylem based on integrated map

Traits	LG	Peak LOD	Co-segregated marker	Peak Location (cM)	*R*^*2*^(%)	Confidence interval (cM)
XY16	2	3.41	chr2_8,632,628	59.32	8.34	49.13–75.07
XY17	2	4.91	chr2_8,632,628	59.32	11.73	57.29–70.99
BLUP	2	4.61	chr2_8,632,628	59.32	9.38	59.32–74.88

*R*^*2*^ represents the individual contribution of one QTL to the variation in cold hardiness

We tried to calculated the best linear unbiased predictor (BLUP) value for each individual line across all environments using the mixed linear model in the R package “lme4” and then the BLUP values were used for QTL mapping based on integrated map (Additional file 9: Data S6). A major QTL related to LTP was identified on LG15, corresponding to the confidence interval of 52.42 cM–68.94 cM, explained 7.33% of the total phenotypic variation (Table 4 and Fig. 4); QTL related to LTX was identified on LG2, corresponding to the confidence interval of 59.32 cM–74.88 cM, explained 9.38% of the total phenotypic variation (Table 5 and Fig. 4).

Confidence interval of PH16 in LG3 and LG15corresponding to the region of Chr3: 6669508-Chr3:7621469 and chr15:15072086-chr15:16909415 on physical map; the QTL region of transformed LTP values corresponding to chr15:15252067- chr15:17430019 on physical map (Table 4). QTL confidence intervals of XY16 and XY17 were both located on LG2, corresponding to the physical map region of Chr2:6750680-Chr2:17798856 and chr2:8147811-chr2:16574359; the QTL region of transformed LTX values corresponding to chr2:8632628-chr2:17864890 on physical map (Table 5).

A stable QTL overlapping region was discovered on LG15 between PH16 and the transformed BLUP LTP values (Fig. 4), covering a confidence interval 52.42 cM–68.94 cM with flanked markers chr15_15,252,067 and chr15_16,909,415 corresponding to chr15:15252067-chr15:16909415 on physical map; For LTX, a stable QTL overlapping region was discovered on LG2 between XY16, XY17 and the transformed BLUP LTX values (Fig. 4), covering a confidence interval 59.32 cM–70.99 cM with flanked markers chr2_8,632,628 and chr2_16,574,359, corresponding to chr2: 8632628-chr2: 16574359 on physical map.

A total of 458 genes were selected based on these two overlapping regions on LG2 and LG15 according to their functional annotation registered in the database (Additional file 10: Data S7) and then the gene ontology (GO) enrichment analysis was performed for genes. Finally, 215 genes were classified into 10 significant GO terms (Additional file 11: Fig. S4). Four genes which involved in the GO term “response to cold” (GO: 0009409) were selected as the candidate cold hardiness resistance genes (Table 6).

**Table 6:** Candidate genes related to cane cold hardiness resistance

Gene ID	Physical position	Functional annotation
VIT_02s0033g01120	chr02:16403096–16,408,471	Dehydration-responsive protein
VIT_15s0048g01980	chr15:16098098–16,109,673	COP9 signalosome complex subunit 1
VIT_15s0048g02410	chr15:16591114–16,601,303	Myb CCA1 (circadian clock associated 1)
VIT_15s0048g02700	chr15:16831342–16,836,139	RNA recognition motif (RRM)-containing protein

## Discussion

### Cold hardiness phenotypic determination

In our study, the grapevine cultivar ‘Zuoyouhong’ was came from the cross of *V. vinifera* L. and *V. amurensis* Rupr., and ‘Cabernet sauvignon’ belongs to *V. vinifera* L., crossing of these two cultivars yields a large number of offspring, indicating good performance of interspecific hybridization affinity. Based on our observation, the cold hardiness value of the offspring showed extensive continuous variation and provides an important population material for cold hardiness QTL mapping. Besides that, we also conducted the filed observation of many grapevine cultivars from different species for many years. For grapevine cultivars which belong to *V. vinifera* L., the average value of LTP and LTX were − 21.10 °C and − 31.20 °C; grapevine cultivars which belong to *V. labrusca* L. were − 25.20 °C and − 34.96 °C; grapevine cultivars which belong to *V. amurensis* Rupr. were − 32.85 °C and − 39.68 °C; Cultivars which came from the interspecies cross of *V. vinifera* × *V. amurensis* were − 26.11 °C and − 36.7 °C; cultivars which came from *V. vinifera* × *V. labrusca* were − 22.24 °C and − 32.86 °C. Some researches indicated that *V. amurensis* was the most responsive species to temperature fluctuations and showed strong cold hardiness [55, 56], this was the same to our observation. In this study, cold hardiness was identified by low-temperature exotherm method, which shows much higher accuracy than traditional electrical conductivity method and has been widely utilized in apple, red maple, walnut, grape, and other woody plants [55–61].

### Genetic map evaluation

In recent years, numerous genetic maps have been constructed for grape by using SNP markers based on next-generation sequencing technology. The sequencing strategies included genotyping by sequencing (GBS), specific locus amplified fragment sequencing (SLAF), and restriction site-associated DNA sequencing (RAD), increasing the number of markers from more than one thousand to tens of thousands. This greatly narrowed the average genetic inter-marker distance of linkage maps from 1.32 cM to 0.05 cM [3, 6, 11, 12, 16, 40]. In this study, the hybrid offspring number was 181 and we achieved 322.68Gb sequencing data. SNP marker detection and screening revealed 16,076, 11,643, and 25,917 SNP markers anchored in female, male, and integrated maps with average genetic distances of adjacent makers of 0.65 cM, 0.77 cM, and 0.41 cM, respectively. Based on our integrated map, many markers in close physical proximity were co-segregated in a unique distance locus due to lack of recombination, for example, in LG1, marker chr1_19616688, chr1_19602344, chr1_19606836, chr1_19634588, these four markers were located at the same genetic position in close physical proximity. Considering the population size used in our study, it is necessary for us to use more sequencing individuals in the future and recalculate the location of these markers for further increase the quality of our linkage map.

**Fig. 5:**
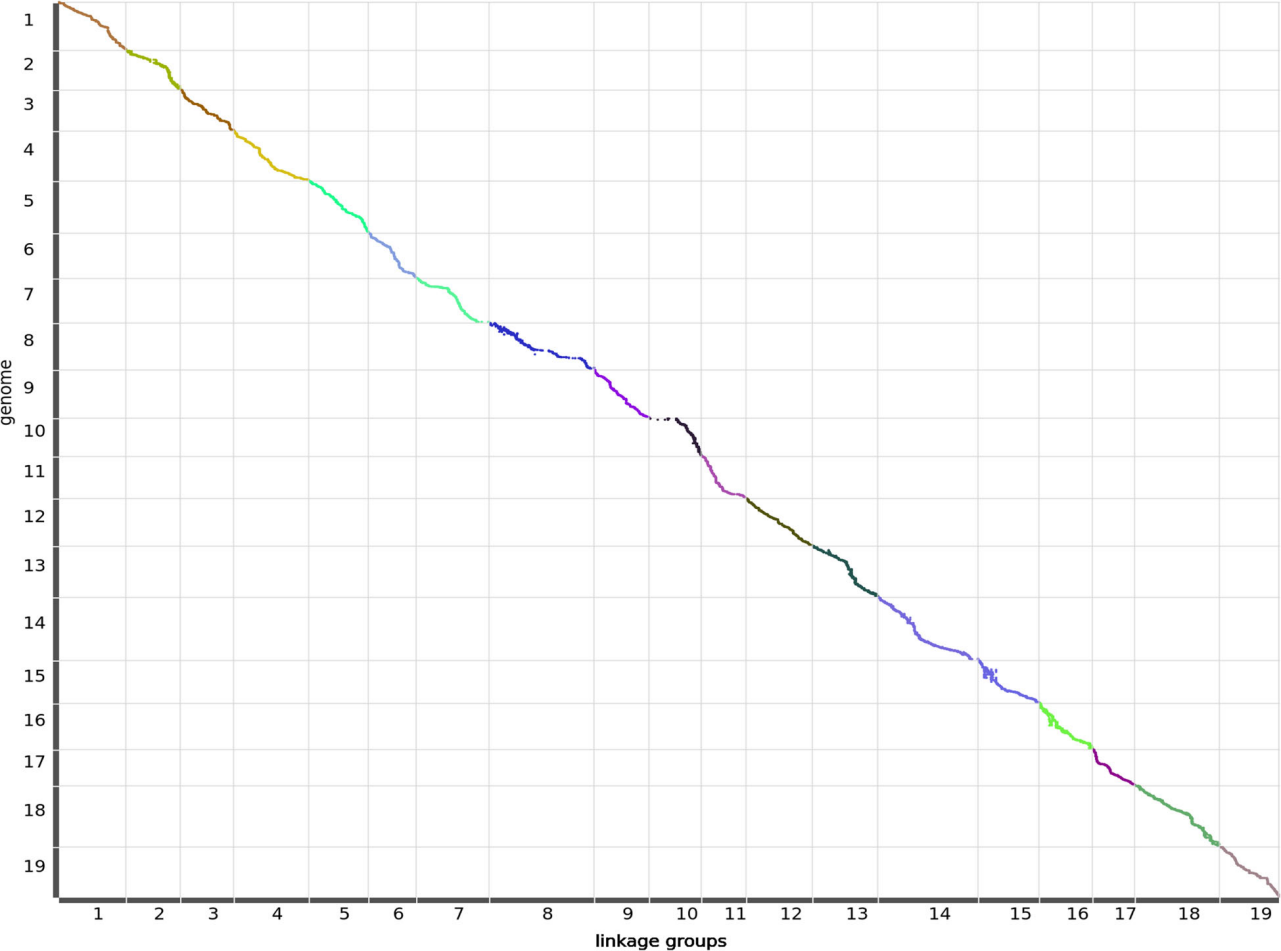
Collinearity analysis between integrated map and referenced grapevine genome PN40024. X-axis represents the genetic length of each linkage group; Y-axis represents the physical length of each linkage group

**Fig. 6:**
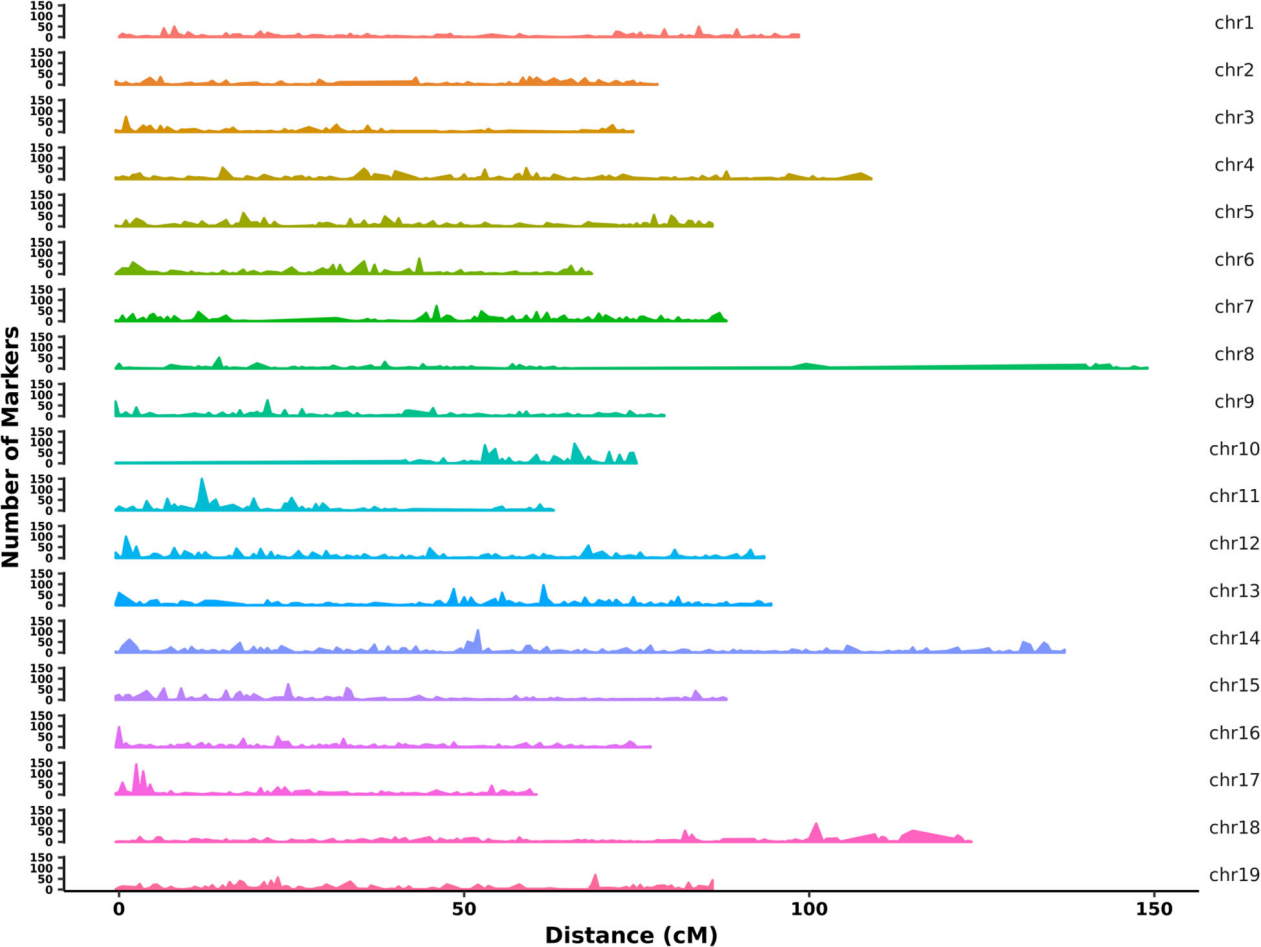
Marker density of integrated map. X-axis represents genetic length of 19 linkage groups. Y-axis represents markers number of each linkage group. The marker number per 0.5 cM of each linkage group was calculated and showed by sliding windows

Collinearity is an important indicator of the quality of a linkage map and is based on the marker order in linkage maps compared to the locations on the reference grapevine genome (Fig. 5). In this study, the Spearman coefficient of most linkage groups was higher than 0.99 (Additional file 12: Data S8), the marker order in the linkage map of our study showed good collinearity performance compared to the physical map, indicating a higher accuracy of genetic recombination rate. This was also observed for the haplotype and heat maps (Additional file 13: Fig. S5 and Additional file 14: Fig. S6). The linkage map in our study also showed good marker densities across the chromosome (Fig. 6). Some larger gaps were observed in LG7, LG8, LG10, and LG14. Previous studies have showed that unlinked scaffolds which account for 9% of the grapevine reference sequence remains unanchored to chromosome and they were called Unknown chromosome [62]. Tello et al. 2019 found that 76 SNP markers from Unknown chromosomes were positioned in eight LGs [13]. While in our study, the “Unknown” chromosome markers were not called. So, markers from Unknown chromosomes may be the reason for larger gaps, because they might be positioned in these regions. While in our study, no unknown chromosome markers were called. The absence of marker polymorphism in these regions, heterozygosity of the parents and missing data can also limit the marker detection and yield large gaps [17, 63–65]. Zhu et al., 2018 also found that long physical distance can also related to large gaps [12]. In our study, flanking markers chr10_138837, chr10_714452, which located on genetic distance 0.0 cM and 10.3 cM in female map LG10, they were physically locatted13 and 70 kb apart in physical map.

### QTL mapping and candidate genes

The application of molecular markers has greatly promoted the process of grape breeding, but obtaining stable genetic QTLs and applying them in molecular-assisted breeding remains difficult. Based on the phenotypic data of cane cold hardiness and constructed genetic map, we totally found 6 QTLs on integrated map. The explained phenotypic variation was between 7.33 and 11.73%. Overlap regions of QTLs related to LTP and LTX were detected in LG2 and LG15 on the integrated map. The lower explained phenotypic variation in this study was similar to other quantitative traits of grapevine, such as cluster number, single cluster weight, and single berry weight [46, 66]. This may because the metabolic regulatory pathways related to these quantitative traits are complex with large numbers of QTLs controlling a certain trait [51]. According to Beavis effect, when the sample size was small, the explained phenotypic variation would be greatly inflated and the larger the sample size, the smaller of the QTL effect and the closer to the true value [67–69].

According to the previous studies, candidate genes involved in the stable QTL regions may play important role in grapevine resistance to cold hardiness. Dehydration-responsive protein contains cis-acting element DRE (dehydration-responsive element), in *Arabidopsis*, it can be regulated by dehydration-responsive element binding protein through abscisic acid (ABA)-independent pathway under cold and dehydration environment [70]. The COP9 signalosome (CSN) is a multiprotein complex that is conserved in most eukaryotes. Cold stress rapidly induces the expression of a series of cold-regulated (COR) genes in *Arabidopsis* and the expression of COR genes requires the activities of CSN [71, 72]. It has been reported that a number of RNA-binding proteins from a cyanobacterium comprise a single RRM module and are highly expressed in response to cold stress [73, 74]. A MYB-like gene *VaAQUILO* from amur grape (*V. amurensis* Rupr.) was induced by cold, overexpression results of this gene in transgenic *Arabidopsis* and in *Amur* grape calli showed significantly tolerance to cold [75]. The identification of the most relevant genes would provide some references for understanding the molecular mechanisms operating in grapevine cold hardiness. However, these genes were speculative and functional validations would be necessary to understand the molecular mechanism in the future. Finally, this study was first to localize two major reproducible QTLs and can provide important reference for grapevine cold hardiness breeding.

## Conclusion

Compared to previously constructed genetic maps, the SNP marker number in the in this study was much higher and we obtained a high-density and high-quality genetic map, providing a foundation for fine QTL mapping of important agronomic traits of grapevine. We first identified six QTLs associated with grape cane phloem and xylem cold hardiness. Based on the preliminary QTL mapping results, we detected four candidate genes that may be involved in regulating cold hardiness in grapevine. Until now, there are no reports of grape cold hardiness QTL mapping, thus, these results will provide a reference for future studies, and we will continue to conduct grapevine cold hardiness research based on our results in combination with other biological technologies such as transcriptomics, proteomics, and metabolomics approaches. The results in our study will provide an important theoretical basis for grapevine cold hardiness breeding.

## Methods

### Plant material

In this study, the grape cultivar ‘Cabernet sauvignon’ (*V. vinifera* L.) and ‘Zuoyouhong’ (*V. vinifera* × *V. amurensis*) was planted in the vineyard of Shenyang Agricultural University (E123°24′, N41°50′, 55 m above sea level). Crossbreeding work was conducted based on the maternal parent ‘Cabernet sauvignon’ and paternal parent ‘Zuoyouhong’ in May 2013 and the seeds were collected in October 2013. The collected seeds were stored under a stratification method between October 2013 and February 2014 and sown in the greenhouse of the same vineyard in March 2014. In total, 680 individuals were harvested and 181 seedlings and the two parents planted in green houses were used as the mapping population.

### Cane phloem and xylem cold hardiness identification

**Fig. 7:**
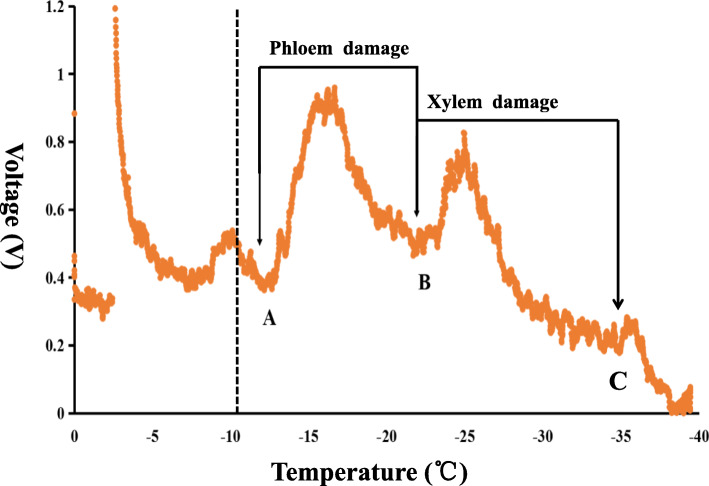
The lethal temperatures of phloem and xylem parenchyma death detected by using low-temperature exotherm method. Site B represents the lethal temperature of phloem, site C represents the lethal temperature of xylem

The low-temperature exotherm method was used to identify cold hardiness. Annual grapevine canes were collected in midwinter of 2016, 2017 and 2018 for 181 genotypes and two parents and then they were kept in cold room with 4 °C. After that, the cold hardiness detection was conducted. Approximately 30-mm long cylindrical sections of cane were cut from the internode area between nodes four and eight, and two such cane pieces were each placed directly on thermoelectric modules (TEM) that can convert the thermal signals to voltage (mV) outputs. Three replicates per genotype were detected. To ensure the adequate contact between the tissues and TEM, foam insulation pads were used and placed on the top of can pieces in each well and then covered with a chamber lid. A total of three chambers (containing 27 TEMS) were stacked in a programmed cooling refrigerator (Anke Environmental Testing Equipment Company, Hefei, China) and the program was as follows: temperature was decreased from room temperature to 4 °C held at this temperature for 1 h; the temperature was decreased from 4 °C to − 40 °C at a rate of 4 °C /h, and then held for 1 h and return to room temperature over 1 h. The ultra-low temperature exothermic process of plant cells during the stable temperature drop was recorded in every 15 s with a data acquisition and processing system (Beijing Huayi Ruike Technology, Beijing, China). For canes, the lethal temperatures of phloem (LTP) were determined by assuming the first range (from site A to site B) of exotherms below − 10 °C. As the temperature continued to decline, a second range of exotherms (from site B to site C) indicated the xylem parenchyma death (LTX) (Fig. 7). In this study, the temperature values at site B and C were recognised as the lethal temperature of phloem and xylem, and these values were used for grapevine cold hardiness evaluation.

### Library construction for sequencing

Young and healthy leaves were collected from the two parents and 181 individuals. The leaves were frozen in liquid nitrogen and stored at − 80 °C. A modified CTAB method was used to extract genomic DNA [76]. A NanoDrop 2000 spectrophotometer (Thermo Fisher Scientific, Waltham, MA, USA) was used to evaluate DNA concentration and quality. The qualified DNA of each individual was digested by TaqI restriction endonuclease (New England Biolabs, Ipswich, MA, USA) and then barcoded P1 adapters were ligated to the TaqI restriction site for each individual. Thereafter, samples were pooled in proportional amounts for shearing to an average size of 500 bp with a Bioruptor (Diagenode, Liège, Belgium). Sequencing libraries were constructed randomly with a total of 24 samples per library. Fragment sizes ranging from 300 to 500 base pairs were extracted by 2% agarose gel electrophoresis and then ligated with the P2 adapter containing unique Illumina barcodes (San Diego, CA, USA). The constructed library was amplified by PCR with Phusion high-fidelity DNA polymerase (New England Biolabs) and the running conditions were: 98 °C for 2 min, followed by 13 cycles at 98 °C for 30 s, 60 °C for 30 s, and 72 °C for 15 s, and a final extension at 72 °C for 5 min. Finally, the samples of each individual were sequenced on an Illumina HiSeq™ platform with the Illumina PE150 strategy (paired-ends). The clean read data size of the two parents was 10 Gb and the data size of each offspring were 1 Gb (Q30 > 80%).

### SNP marker development and genetic linkage map construction

The original image obtained by Illumina HiSeqTM sequencing was converted into sequence format by base calling, and the obtained original sequence was stored in FASTQ format. The joints were removed from the obtained original data, filtration was carried out to remove low-quality data, and clean reads for subsequent test analysis were obtained. High-quality filtered data were mapped to the *Vitis vinifera* PN40024 reference genome (https://plants.ensembl.org/Vitis_vinifera/Info/Index) by using BWA software and then the BWA file was obtained [77]. Subsequently, the Best Practices pipeline of GATK software was used to calibrate the BWA file and develop SNP markers [78]. Based on basic genetic principles, all SNPS were screened to obtain molecular markers that met the requirements of genetic map construction. The filter standards were as follows: sequencing depths of the two parents and progeny were more than 10×; at least of one of the two parents possessed a heterozygous genotype; the segregation ratio of progeny was tested by Chi-square test (*p* < 0.05); the genotype missing rate was 0. There are five segregation types of CP populations (lm × ll, nn × np, hk × hk, ef × eg, and ab × cd), and they were genotyped into three types: ‘lm × ll’ represents markers with first parent heterozygous and second parent homozygous; ‘nn × np’ represents markers with the first parent homozygous and the second parent heterozygous; ‘hk × hk’ represents markers with both parents heterozygous, these three segregation types were used for constructing male, female, and integrated linkage map. Finally, JoinMap 5.0 software was used to construct the genetic linkage map, and the minimum logarithm of odds (LOD) score of 7.0 was used to establish linkage groups. Map distances (cM) were converted using recombination frequencies through the Kosambi mapping function. The visualised linkage maps were subsequently drawn using MapChart 2.2 [79].

### Cold hardiness QTL mapping and candidate gene identification

The LTP and LTX (mean value of three replicates per genotype) in 2016, 2017, 2018 and the transformed phenotypic values were used separately for QTL mapping. Interval mapping (IM) and Multiple QTL mapping (MQM) method (four-way cross format) were used for integrated map based on the R/qtl package [80]. Genome wide (GW) LOD threshold (α = 0.5) were established through 1000 permutations of the phenotypic data and used for both IM and MQM QTL detection. QTLs with LOD score higher than GW LOD threshold were considered as significant ones. Firstly, IM analysis was conducted to find regions with potential QTL effects. Then, scored markers in these regions were used as cofactors in MQM analysis (max.qtl was set to 10 for forward selection). Genome scans were performed with a 1 cM step. Markers which flanked or located at the peak LOD of a QTL were recognised as co-segregated markers. The explained phenotypic variation of each QTL (*R*^*2*^) was estimated using the “fitqtl” function. 1-LOD confidence interval which corresponded to 95% confidence interval was calculated using the “lodint” function [81]. Candidate genes research was conducted based on the confidence interval (CI) of each QTL on integrated map. Genes were selected according to 12X V1 versions (Grape Genome Database, http://genomes.cribi.unipd.it/DATA/). A gene ontology (GO) enrichment analysis was performed for these identified genes using the “singular enrichment analysis” tool in the GO Analysis Toolkit and Database (http://bioinfo.cau.edu.cn/agriGO). The statistical significance of functional enrichment within the intervals was evaluated using the hypergeometric distribution, and the significant of GO terms was *P* < 0.05.

## Supplementary information

**Additional file 1: Data S1.** The phenotypic data used in this study.

**Additional file 2: Figure S1.** Clean read number and average sequencing depth distribution of the 181 hybrid offspring.

**Additional file 3: Data S2.** Markers used for genetic linkage map construction.

**Additional file 4: Data S3.** The Mendelian segregation and depth of each marker.

**Additional file 5: Data S4.** Marker number statistics of female, male and integrated map.

**Additional file 6: Data S5.** Bin markers of female, male and integrated map.

**Additional file 7: Figure S2.** Marker distribution and genetic length in 19 linkage groups of female parent ‘Cabernet sauvignon’.

**Additional file 8: Figure S3.** Marker distribution and genetic length in 19 linkage groups of male parent ‘Zuoyouhong’.

**Additional file 9: Data S6.** BLUP values of LTP and LTX used for QTL mapping.

**Additional file 10: Data S7.** Candidate genes involved in the confidence intercal of QTL mapping.

**Additional file 11: Figure S4.** Gene ontology (GO) enrichment analysis for gnes involved in stable QTL regions.

**Additional file 12: Data S8.** Spearman coefficient and map coverage of integrated map.

**Additional file 13: Figure S5.** Haplotyp maps of integrated map.

**Additional file 14: Figure S6.** Heat maps of integrated map.

## Data Availability

The data supporting the results presented in this article are included as additional files. The raw sequencing data were deposited at the NCBI Sequence Read Archive (SRA) with the accession Number SRP266857.

## Abbreviations

QTL: Quantitative trait loci
RAD: Restriction-site associated DNA sequencing
SNP: Single-nucleotide polymorphism
MAS: Marker-assisted selection
NGS: Next-generation sequencing
GWAS: Genome-wide association study
LTP: Lethal temperature of phloem
LTX: Lethal temperature of xylem
LOD: Logarithm of odds
BLUP: Best linear unbiased predictor
GBS: Genotyping by sequencing
SLAF: Specific locus amplified fragment sequencing
DRE: Dehydration-responsive element
CSN: COP9 signalosome
COR: Cold-regulated genes
IM: Interval mapping
MQM: Multiple QTL mapping
CI: Confidence interval
