# Supplementary figures and images for: High-density genetic linkage map construction and cane cold hardiness QTL mapping for Vitis based on restriction site-associated DNA sequencing
Source: BMC Genomics. 2020 Jun 22;21:419. doi: 10.1186/s12864-020-06836-z (PMC7310074; doi:10.1186/s12864-020-06836-z)

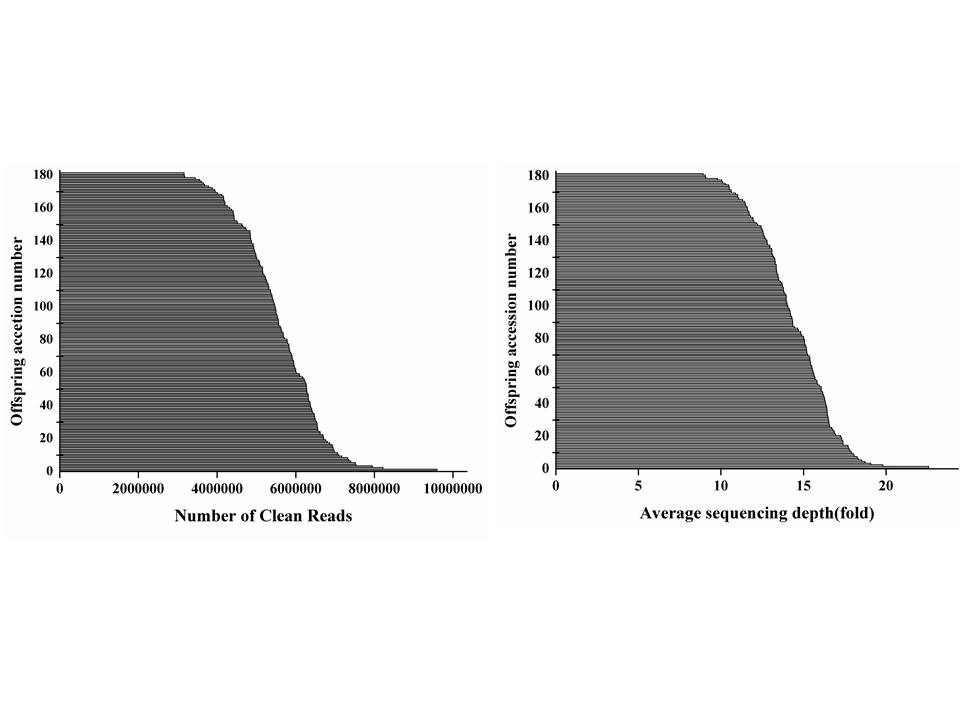

Supplement: Supplementary file 2 — Additional file 2: Figure S1. Clean read number and average sequencing depth distribution of the 181 hybrid offspring. [file 12864_2020_6836_MOESM2_ESM.tif]

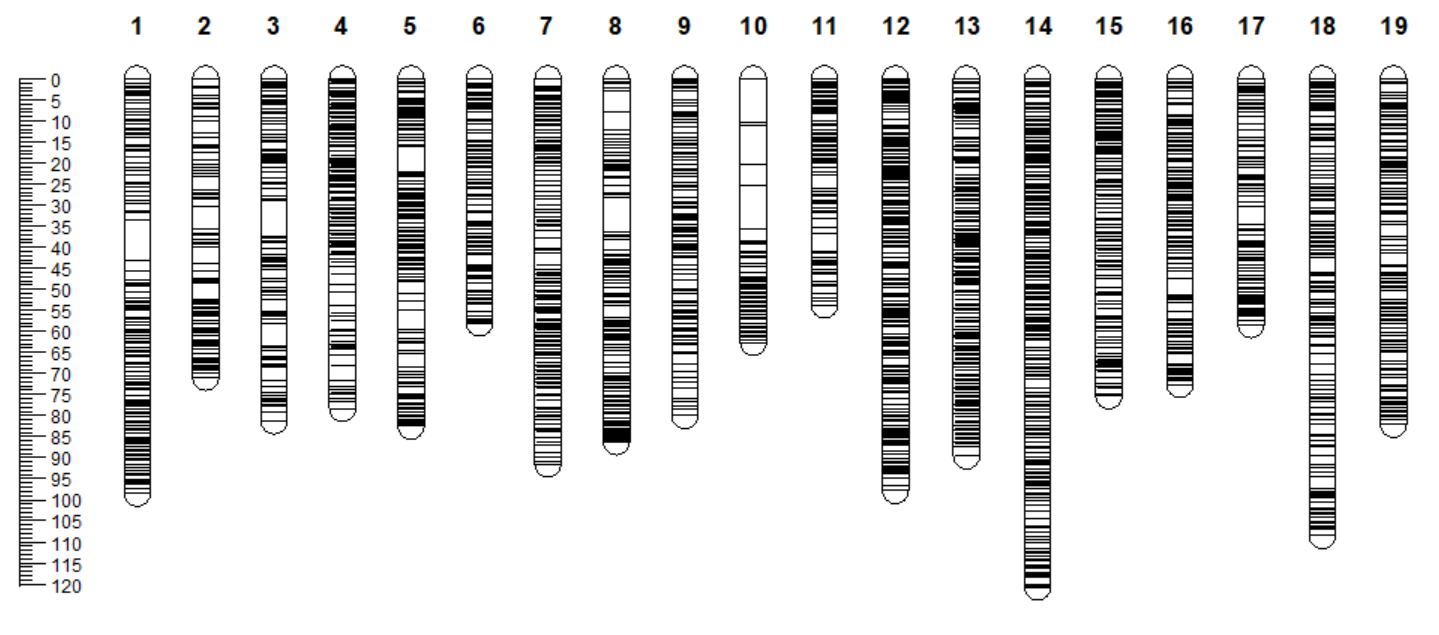

Supplement: Supplementary file 7 — Additional file 7: Figure S2. Marker distribution and genetic length in 19 linkage groups of female parent ‘Cabernet sauvignon’. [file 12864_2020_6836_MOESM7_ESM.png]

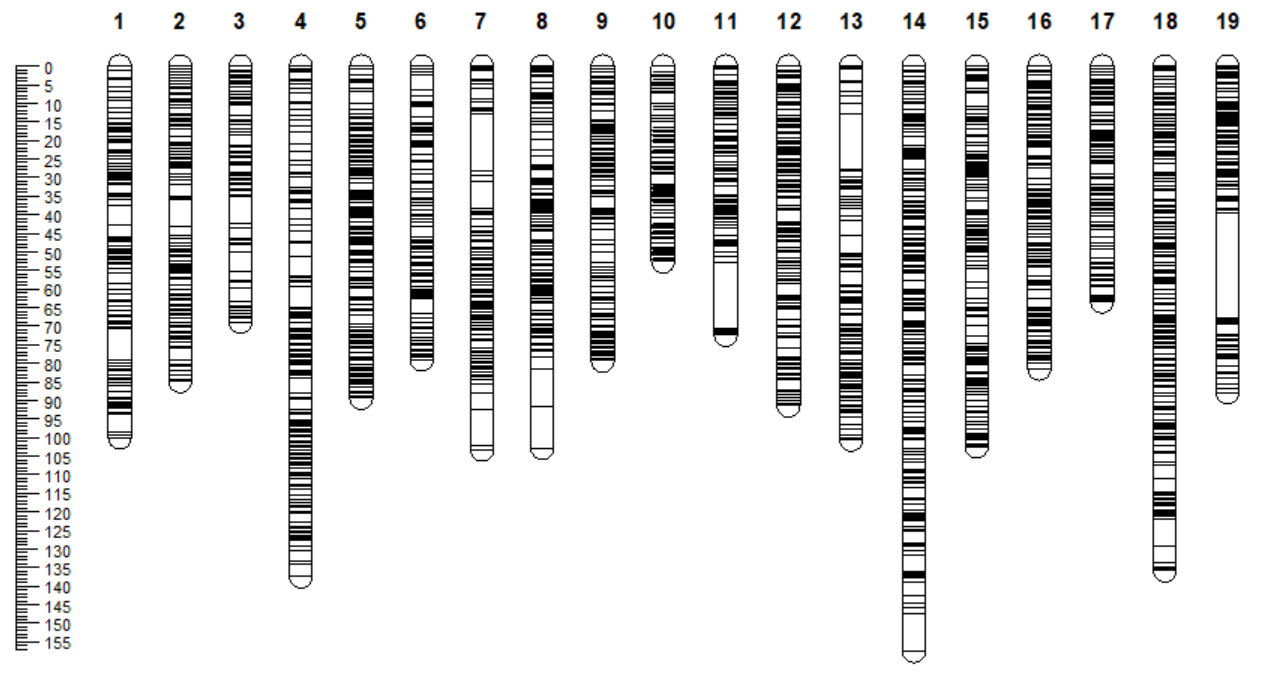

Supplement: Supplementary file 8 — Additional file 8: Figure S3. Marker distribution and genetic length in 19 linkage groups of male parent ‘Zuoyouhong’. [file 12864_2020_6836_MOESM8_ESM.png]

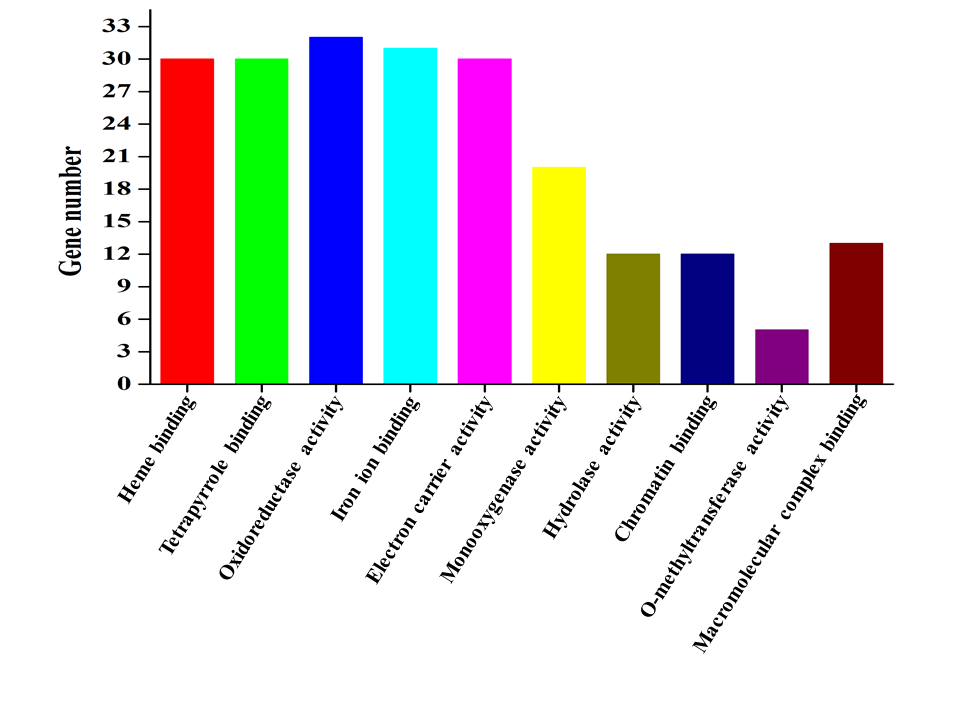

Supplement: Supplementary file 11 — Additional file 11: Figure S4. Gene ontology (GO) enrichment analysis for gnes involved in stable QTL regions. [file 12864_2020_6836_MOESM11_ESM.tif]

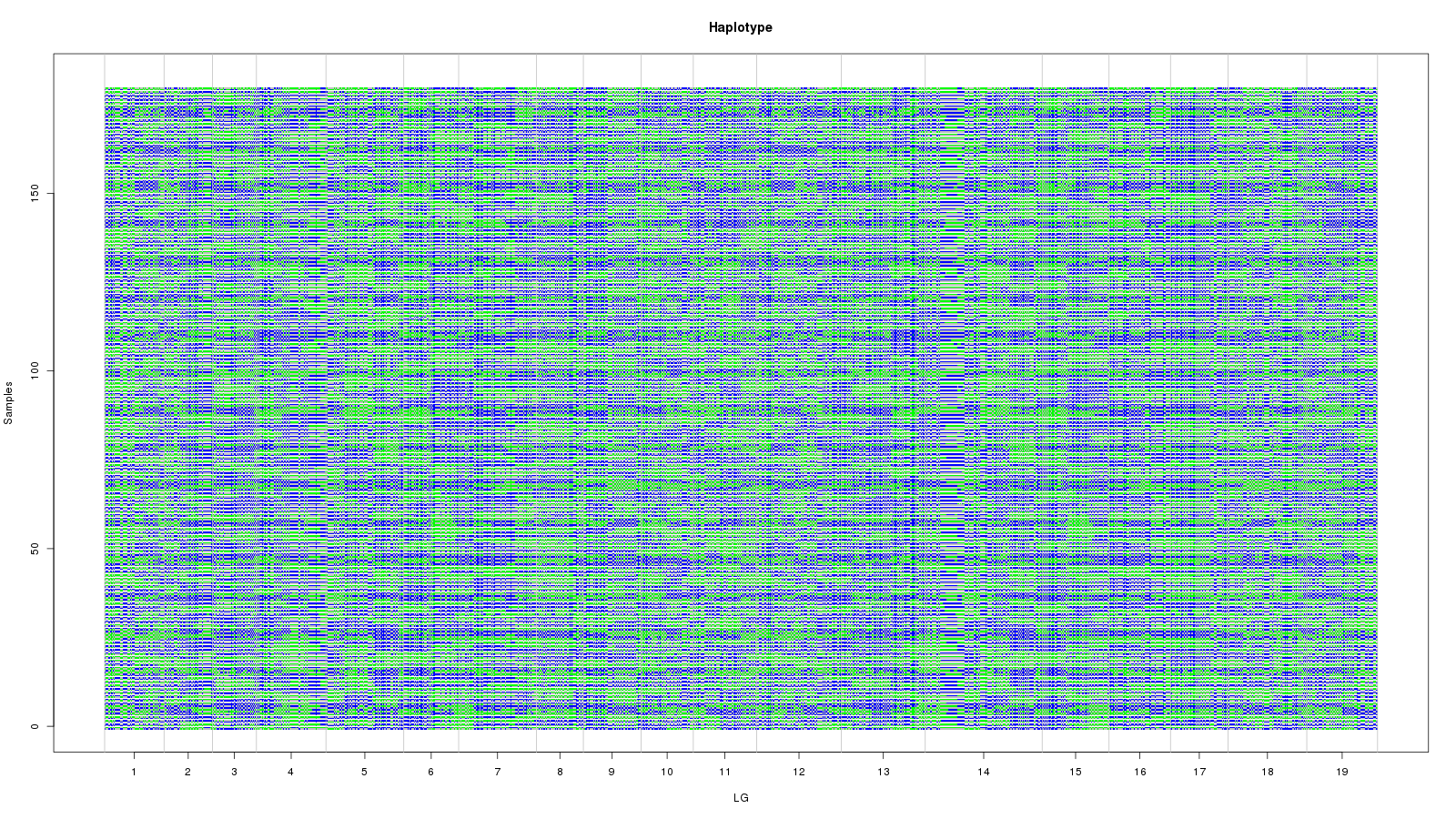

Supplement: Supplementary file 13 — Additional file 13: Figure S5. Haplotyp maps of integrated map. [file 12864_2020_6836_MOESM13_ESM.png]

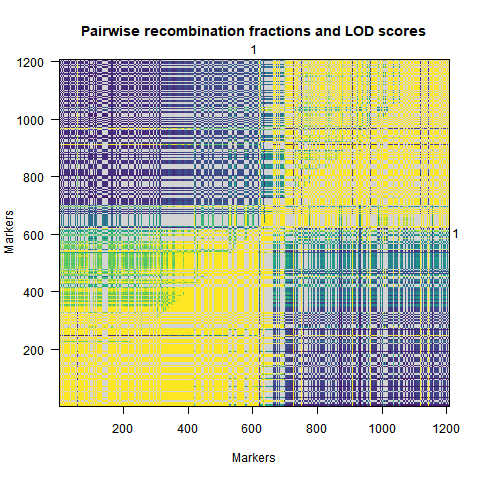

Supplement: Supplementary file 14 — Additional file 14: Figure S6. Heat maps of integrated map. [file 12864_2020_6836_MOESM14_ESM.zip › Additional file 3. Heat maps/1.heatMap.sexAver.png]

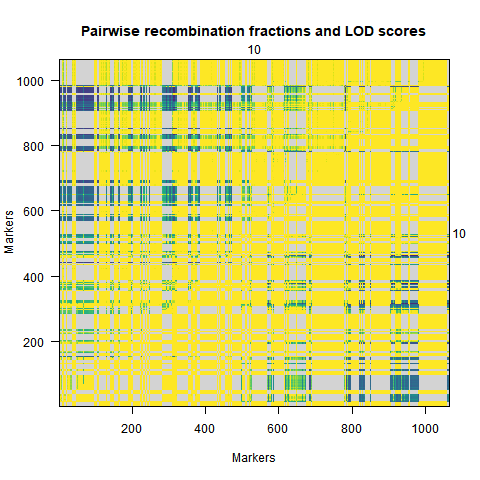

Supplement: Supplementary file 14 — Additional file 14: Figure S6. Heat maps of integrated map. [file 12864_2020_6836_MOESM14_ESM.zip › Additional file 3. Heat maps/10.heatMap.sexAver.png]

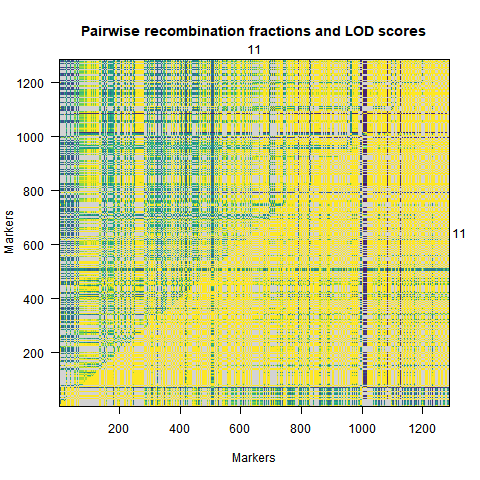

Supplement: Supplementary file 14 — Additional file 14: Figure S6. Heat maps of integrated map. [file 12864_2020_6836_MOESM14_ESM.zip › Additional file 3. Heat maps/11.heatMap.sexAver.png]

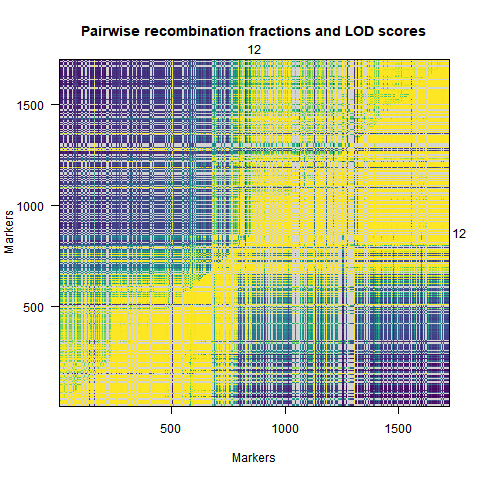

Supplement: Supplementary file 14 — Additional file 14: Figure S6. Heat maps of integrated map. [file 12864_2020_6836_MOESM14_ESM.zip › Additional file 3. Heat maps/12.heatMap.sexAver.png]

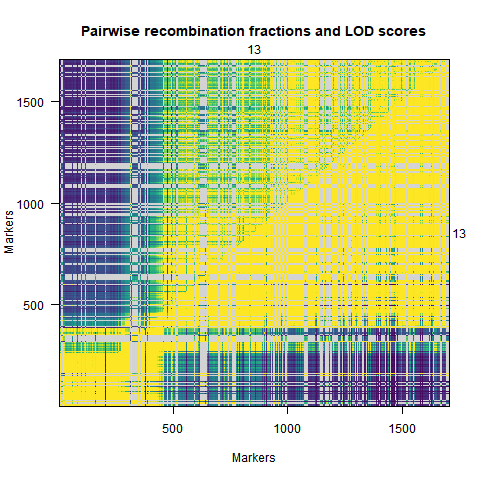

Supplement: Supplementary file 14 — Additional file 14: Figure S6. Heat maps of integrated map. [file 12864_2020_6836_MOESM14_ESM.zip › Additional file 3. Heat maps/13.heatMap.sexAver.png]

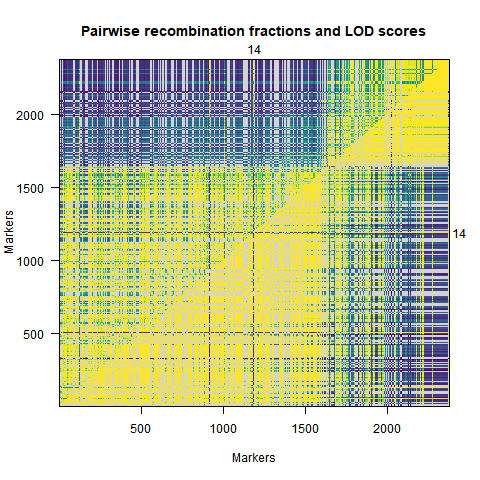

Supplement: Supplementary file 14 — Additional file 14: Figure S6. Heat maps of integrated map. [file 12864_2020_6836_MOESM14_ESM.zip › Additional file 3. Heat maps/14.heatMap.sexAver.png]

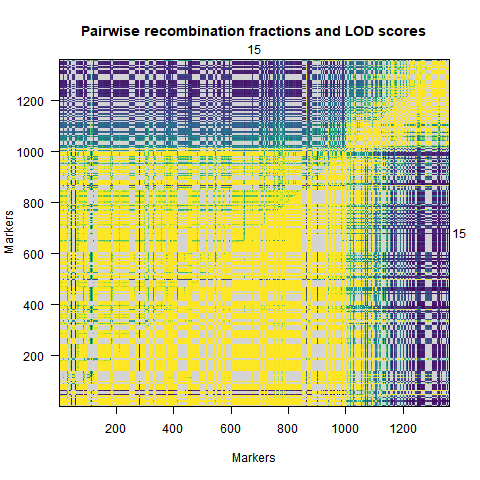

Supplement: Supplementary file 14 — Additional file 14: Figure S6. Heat maps of integrated map. [file 12864_2020_6836_MOESM14_ESM.zip › Additional file 3. Heat maps/15.heatMap.sexAver.png]

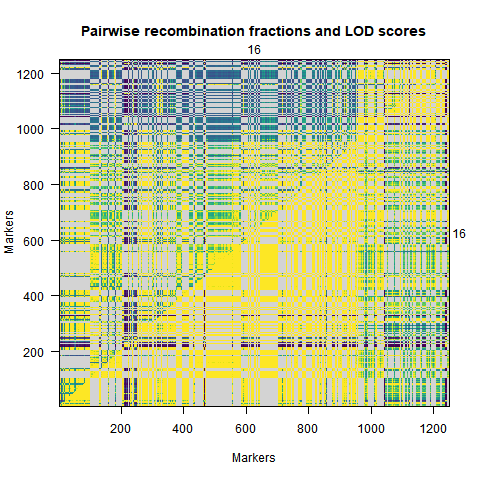

Supplement: Supplementary file 14 — Additional file 14: Figure S6. Heat maps of integrated map. [file 12864_2020_6836_MOESM14_ESM.zip › Additional file 3. Heat maps/16.heatMap.sexAver.png]

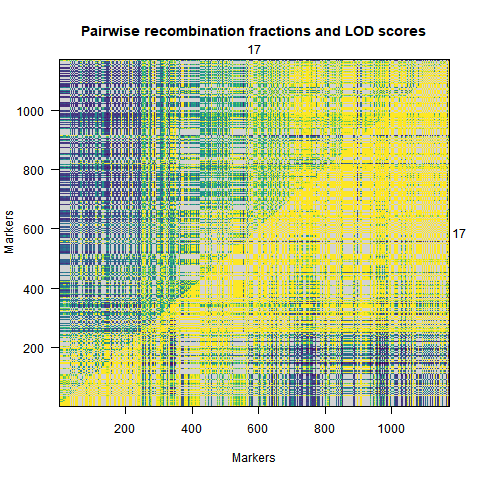

Supplement: Supplementary file 14 — Additional file 14: Figure S6. Heat maps of integrated map. [file 12864_2020_6836_MOESM14_ESM.zip › Additional file 3. Heat maps/17.heatMap.sexAver.png]

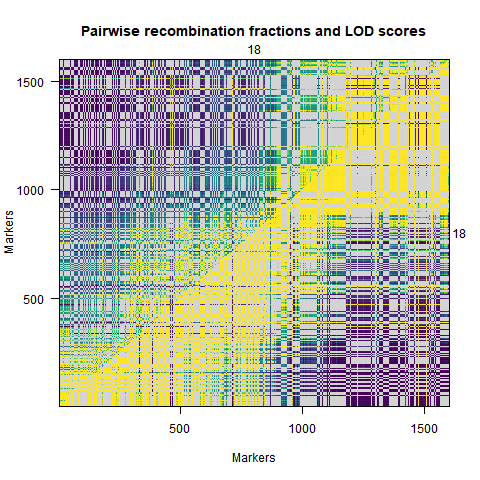

Supplement: Supplementary file 14 — Additional file 14: Figure S6. Heat maps of integrated map. [file 12864_2020_6836_MOESM14_ESM.zip › Additional file 3. Heat maps/18.heatMap.sexAver.png]

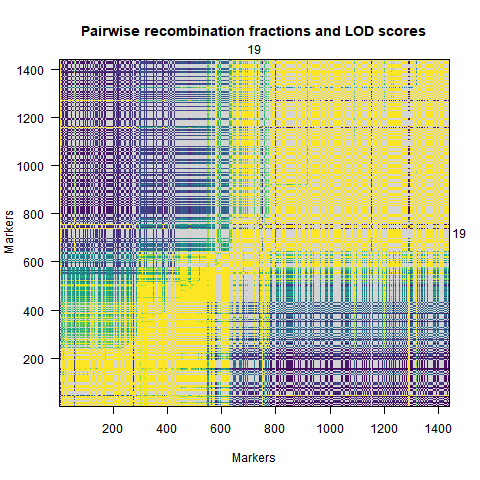

Supplement: Supplementary file 14 — Additional file 14: Figure S6. Heat maps of integrated map. [file 12864_2020_6836_MOESM14_ESM.zip › Additional file 3. Heat maps/19.heatMap.sexAver.png]

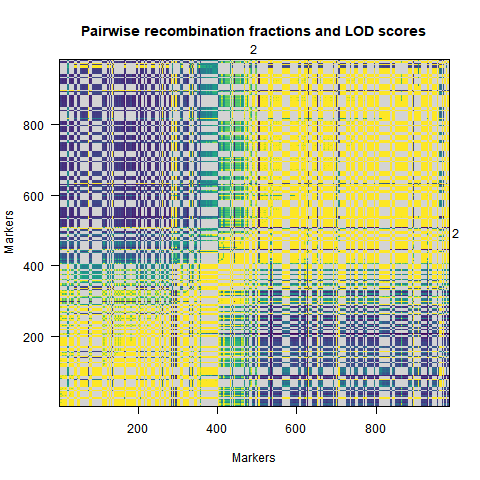

Supplement: Supplementary file 14 — Additional file 14: Figure S6. Heat maps of integrated map. [file 12864_2020_6836_MOESM14_ESM.zip › Additional file 3. Heat maps/2.heatMap.sexAver.png]

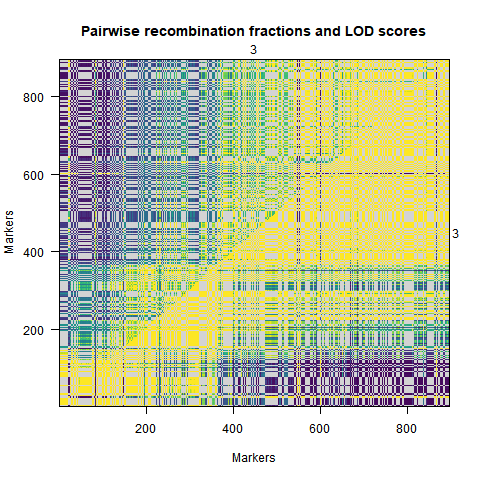

Supplement: Supplementary file 14 — Additional file 14: Figure S6. Heat maps of integrated map. [file 12864_2020_6836_MOESM14_ESM.zip › Additional file 3. Heat maps/3.heatMap.sexAver.png]

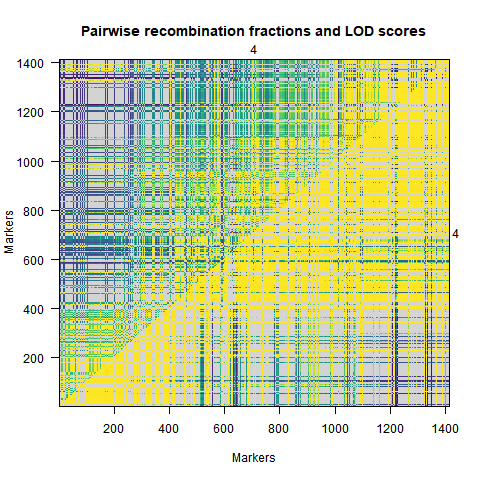

Supplement: Supplementary file 14 — Additional file 14: Figure S6. Heat maps of integrated map. [file 12864_2020_6836_MOESM14_ESM.zip › Additional file 3. Heat maps/4.heatMap.sexAver.png]

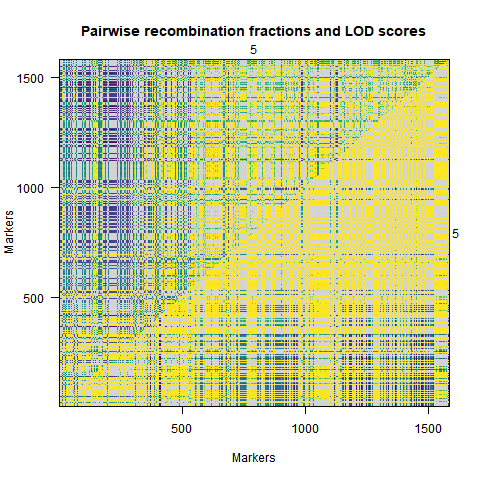

Supplement: Supplementary file 14 — Additional file 14: Figure S6. Heat maps of integrated map. [file 12864_2020_6836_MOESM14_ESM.zip › Additional file 3. Heat maps/5.heatMap.sexAver.png]

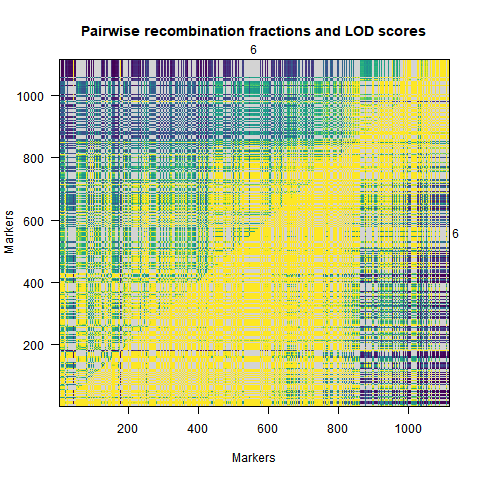

Supplement: Supplementary file 14 — Additional file 14: Figure S6. Heat maps of integrated map. [file 12864_2020_6836_MOESM14_ESM.zip › Additional file 3. Heat maps/6.heatMap.sexAver.png]

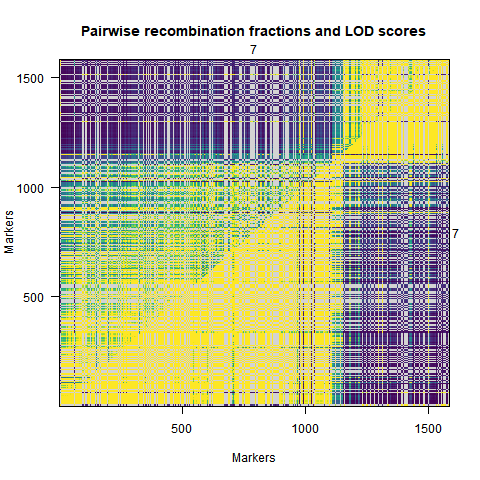

Supplement: Supplementary file 14 — Additional file 14: Figure S6. Heat maps of integrated map. [file 12864_2020_6836_MOESM14_ESM.zip › Additional file 3. Heat maps/7.heatMap.sexAver.png]

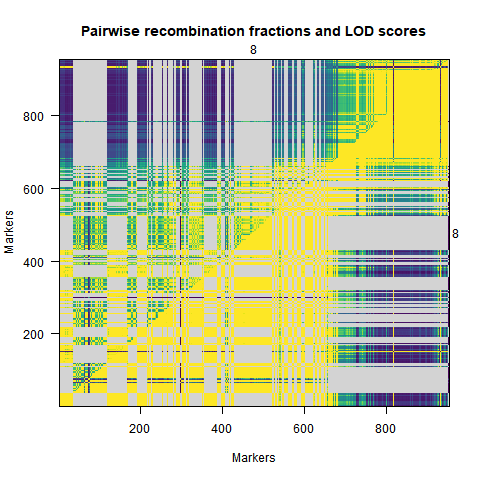

Supplement: Supplementary file 14 — Additional file 14: Figure S6. Heat maps of integrated map. [file 12864_2020_6836_MOESM14_ESM.zip › Additional file 3. Heat maps/8.heatMap.sexAver.png]

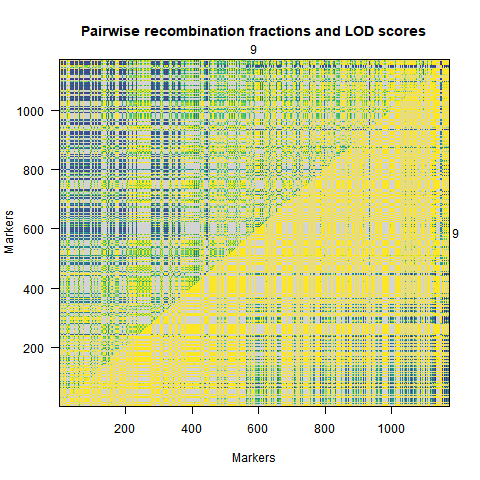

Supplement: Supplementary file 14 — Additional file 14: Figure S6. Heat maps of integrated map. [file 12864_2020_6836_MOESM14_ESM.zip › Additional file 3. Heat maps/9.heatMap.sexAver.png]
