# Supplementary material for: High-density genetic linkage map construction and cane cold hardiness QTL mapping for Vitis based on restriction site-associated DNA sequencing
Source: BMC Genomics. 2020 Jun 22;21:419. doi: 10.1186/s12864-020-06836-z (PMC7310074; doi:10.1186/s12864-020-06836-z)
Supplement: Supplementary file 12 — Additional file 12: Data S8. Spearman coefficient and map coverage of integrated map. [file 12864_2020_6836_MOESM12_ESM.docx]

Table 5 Spearman coefficient and map coverage of integrated map

| LG ID | Spearman coefficient | Physical map length(bp) | Map coverage |
| --- | --- | --- | --- |
| LG1 | 0.99 | 23,037,639 | 99.66% |
| LG2 | 0.99 | 18,779,844 | 99.22% |
| LG3 | 0.99 | 19,341,862 | 99.78% |
| LG4 | 0.99 | 23,867,706 | 99.24% |
| LG5 | 0.99 | 25,021,643 | 99.95% |
| LG6 | 0.99 | 21,508,407 | 99.93% |
| LG7 | 0.99 | 21,026,613 | 99.52% |
| LG8 | 0.99 | 22,385,789 | 99.56% |
| LG9 | 0.99 | 23,006,712 | 99.84% |
| LG10 | 0.99 | 18,140,952 | 99.84% |
| LG11 | 0.99 | 19,818,926 | 99.75% |
| LG12 | 0.99 | 22,702,307 | 99.12% |
| LG13 | 0.99 | 24,396,255 | 99.87% |
| LG14 | 0.99 | 30,274,277 | 99.52% |
| LG15 | 0.99 | 20,304,914 | 99.41% |
| LG16 | 0.99 | 22,053,297 | 99.96% |
| LG17 | 0.99 | 17,126,926 | 99.88% |
| LG18 | 0.99 | 29,360,087 | 99.75% |
| LG19 | 0.99 | 24,021,853 | 99.92% |
